# Right ventricular free wall longitudinal strain during weaning from mechanical ventilation using high-flow or conventional oxygen treatment: a pilot study

**DOI:** 10.1186/s13089-024-00358-5

**Published:** 2024-02-27

**Authors:** Eleni Xourgia, Apostolos Koronaios, Anastasia Kotanidou, Ilias I. Siempos, Christina Routsi

**Affiliations:** 1 First Department of Critical Care Medicine and Pulmonary Services, National and Kapodistrian University of Athens, Medical School Evangelismos Hospital, 45-47 Ipsilantou Street, 10676 Athens, Greece; 2 Department of Heart Surgery, Lnselspital, University Hospital, University of Bern, Bern, Switzerland; 3 Division of Pulmonary and Critical Care Medicine, Department of Medicine, Weill Cornell Medicine, New York, NY USA

**Keywords:** Weaning from prolonged mechanical ventilation, Tracheostomy, High-flow oxygen treatment, Speckle-tracking echocardiography, Right ventricular free wall longitudinal strain

**To the Editor,**

Previous studies on cardiovascular system alterations due to transition from positive to negative intrathoracic pressure because of withdrawal of mechanical ventilation (MV) and resumption of spontaneous breathing focused on left ventricular function [1–3]. Relevant studies on right ventricular (RV) function are scarce [4–6]. Advancements in echocardiography, namely two-dimensional speckle-tracking echocardiography, provide novel parameters, such as RV free wall longitudinal strain (RVFWSL) that may outperform conventional measurements [7]. There is no information on RVFWSL during weaning from MV. We investigated the effect of spontaneous breathing trial (SBT), supported by high-flow oxygen treatment (HFOT), or by T-piece, on RV function in patients with prolonged MV.

Nine tracheostomized patients on MV (assist controlled mode) underwent a 30-min SBT receiving oxygen either via T-piece, or by HFOT, through tracheostomy with a flow rate of 60 L/min, followed by a washout period of 15 min on MV and 30 min with the other modality in a randomized crossover manner. Transthoracic echocardiography was performed on MV (baseline) and at the end of each SBT. Images were acquired in cine-loop format from several consecutive beats and analyzed offline (EchoPAC Version 204; GE Healthcare, Chicago, IL). A repeated-measures analysis of variance (ANOVA) was used to compare echocardiographic and physiological parameters during MV, HFOT, and T-piece. All statistical tests were two tailed; significance was defined as *p* < 0.05.

Fourteen SBT sessions were completed; five patients were studied on two different days with alternative sessions and four patients once. Three patients were successfully weaned from MV (Table 1). There was no significant difference in RVFWSL values measured during MV, HFOT, and T-piece (22.3%, 25.0%, and 24.7%, respectively, *p* = 0.415). Higher baseline RVFWSL values were associated with successful weaning (25.9 vs 20.4%, *p* = 0.045, Table 2).

**Table 1:** Baseline characteristics and outcomes of the included patients

Pt. no.	Age	Admission diagnosis	Days on MV	SOFA score*	Baseline RVFWSL, % (day 1; day 2)**	Baseline PEEP, cmH_2_O (day 1; day 2)	SBT outcome (day 1; day 2)	Weaning outcome (day 1; day 2)***
1	64	Traumatic brain injury	24	5	25.9; 25.6	7; 5	S; S	S; S
2	73	Brain hemorrhage	26	3	20.4; 21.8	5; 5	S; S	F; F
3	71	Lobectomy	26	5	18.0; 20.0	6; 7	S; S	F; F
4	57	Brain hemorrhage	15	3	29.7; 34.1	7; 7	S; S	S; S
5	48	Multitrauma	18	5	18.8; NA	6; NA	S; NA	F; NA
6	47	Multitrauma	60	5	22.8; NA	6; NA	S; NA	F; NA
7	60	Pneumonia	21	4	17.9; NA	4; NA	S; NA	F; NA
8	61	Esophagectomy	32	4	25.9; 28.0	6; 6	S; S	F; F
9	64	Brain hemorrhage	26	6	21.1; NA	6; NA	S; NA	S; NA

MV: mechanical ventilation; SOFA: Sequential Organ Failure Assessment; RVFWSL: right ventricular free wall longitudinal strain; PEEP: positive end-expiratory pressure; SBT: spontaneous breathing trial; NA: not available/applicable; S: successful; F: failed

^*^ On the day of first SBT

^**^ RVFWS values are presented as absolute values, with higher numbers signifying better cardiac function

^***^ Weaning success was defined as the ability of the patient to tolerate spontaneous ventilation through tracheostomy during the next 24 h

**Table 2:** Echocardiographic and physiological parameters of included patients undergoing 14 sessions of spontaneous breathing trial

Variable	Mechanical ventilation	HFOT	T-piece	*p* value*
RVFWSL, %	22.3 (19.7–26.4)	25.0 (21.2–27.8)	24.7 (21.7–28.1)	0.415
TAPSE, mm	22.1 (18.7–24.4)	23.2 (20.3–26.0)	22.1 (19.6–24.0)	0.088
S’ velocity, cm/s	11.0 (8.8–13.0)	13.0 (9.8–13.0)	11.5 (9.8–13.0)	0.214
FAC, %	42.5 (37.0–46.5)	42.0 (37.8–45.3)	42.5 (36.0–45.5)	0.827
RVD_1_, cm	3.7 (3.0–4.0)	3.5 (2.9–4.0)	3.7 (2.9–4.1)	0.174
RVD_2_, cm	2.9 (2.8–3.3)	3.0 (2.9–3.2)	3.0 (2.9–3.3)	0.408
RVD_3_, cm	5.8 (4.3–6.8)	5.8 (4.3–6.9)	6.2 (4.2–6.7)	0.742
RA-R,%	30.0 (24.0–38.8)	35.5 (25.0–43.0)	36.5 (27.8–43.5)	**0.004**^a^
RA volume, ml	35.5 (30.0–48.8)	36.5 (33.3–44.5)	37.0 (33.0–48.3)	0.708
EF, %	50.5 (42.8–55.5)	54.0 (47.5–58.3)	50.5 (45.7–56.5)	**0.006**^b^
SAP, mmHg	143.0 (124.0–153.0)	143.5 (131.0–162.2)	143.5 (135.8–162.0)	0.137
DAP, mmHg	68.5 (62.5–75.8)	66.5 (60.8–72.3)	68.0 (62.3–71.3)	0.118
Heart rate, bpm	83.5 (79.8–97.0)	86.0 (80.8–96.8)	84.0 (80.0–97.3)	0.274
PaO_2_/FiO_2_	280.1 (219.9–333.1)	265.0 (200.0–311.1)	295.0 (207.1–330.0)	0.102
PaCO_2_, mmHg	36.0 (31.8–38.5)	35.5 (33.0–39.3)	35.5 (33.8–37.3)	0.202
ScvO_2_, %	68.4 (61.5–74.3)	73.9 (66.4–75.7)	71.2 (68.3–74.4)	** < 0.001**^c^
*V*_T_, ml §	498.5 (476.3–529.8)	457.0 (396.0–608.3)	485.0 (379.5–588.8)	0.786
Respiratory *f*, bpm #	21.0 (17.5–24.5)	27.0 (23.5–30.0)	29.0 (26.0–32.0)	** < 0.001**^d^
Respiratory *f*/*V*_T_, bpm/L #	41.0 (34.6–55.8)	57.3 (43.6–67.7)	61.9 (46.9–76.7)	**0.004**^e^
PEEP, cmH_2_O	6.0 (5.0–7.0)			
Pplateau, cmH_2_O	16.5 (14.5–18.8)			
Pdriving, cmH_2_O	10.5 (8.5–11.8)			

SBT: spontaneous breathing trial; HFOT: high-flow oxygen treatment; RVFWSL: right ventricular free wall longitudinal strain; TAPSE: tricuspid annular plane systolic excursion; S′ velocity: tricuspid annular peak systolic velocity; FAC, right ventricular fractional area change; RVD_1_, right ventricular basal dimension; RVD_2_: right ventricular midcavity dimension; RVD_3_: right ventricular longitudinal dimension; RA-R: right atrial reservoir strain; SAP: systolic arterial pressure; DAP: diastolic arterial pressure; E: left ventricular ejection fraction; PaO_2_: partial pressure of arterial oxygen; FiO_2_: fraction of inspired oxygen; PaCO_2_: partial pressure of arterial carbon dioxide; ScvO_2_: central venous oxygen saturation; *V*_T_: tidal volume; *f*: frequency; PEEP: positive end-expiratory pressure

^*^For statistically significant differences, pairwise comparisons using Bonferroni correction were performed between the

three groups, with significant results presented as footnotes of this table

^**^ RVFWS values are presented as absolute values. Higher RVFWS values signify better cardiac function

^§^ Via a Wright’s spirometer

^#^ Measured at 2 min after disconnection from mechanical ventilation

^a^Mechanical ventilation vs HFOT (*p* = 0.036) and mechanical ventilation vs T-piece (*p* = 0.047),

^b^mechanical ventilation vs HFOT (*p* = 0.009),

^c^mechanical ventilation vs HFOT (*p* = 0.006) and mechanical ventilation vs T-piece (*p* = 0.011),

^d^mechanical ventilation vs HFOT (*p* = 0.002), mechanical ventilation vs T-piece (*p* < 0.001), and HFOT vs T-piece (*p* = 0.023),

^e^mechanical ventilation vs T-piece (*p* = 0.037)

Our finding of a well-preserved RV response to successful SBTs, as opposed to MV, is consistent with earlier reports using pulmonary artery catheterization [4–6]. Since positive pressure ventilation and particularly positive end-expiratory pressure (PEEP), in most situations, reduce venous return and may increase pulmonary vascular resistance (i.e., RV afterload) [3], one would expect that MV or a modality that applies PEEP could adversely affect the RV function, compared to spontaneous breathing. Nevertheless, this may occur when PEEP induces lung overdistention [8] or in the presence of RV dysfunction [9]. We found no difference in RVFWSL between T-piece and MV, possibly due to the low level of PEEP applied during MV (Table 2). Also, we found no difference in RVFWSL between T-piece and HFOT. Although HFOT via nasal cannula generates some PEEP, HFOT via tracheostomy possibly provides lower degree of PEEP, even with the highest flow [10, 11], attributable to the fact that tracheostomy bypasses the upper airways [11, 12]. Thus, the effect of HFOT via tracheostomy on RV seems negligible. On the contrary, our finding that higher baseline RVFWSL values were associated with successful weaning suggests a role of RV function in weaning outcome. Finally, our finding that right atrial reservoir strain increased after discontinuation of MV (Table 2) possibly signifies improvement of right atrial filling secondary to decreased intrathoracic pressure.

In summary, in tracheostomized patients with prolonged MV, RVFWSL was maintained during successful SBTs, supported by HFOT or T-piece. Higher baseline RVFWSL values were associated with successful weaning.

## Data Availability

The datasets used and/or analyzed during the current study are available from the corresponding author on request.

## Abbreviations

HFOT: High-flow oxygen treatment
RV: Right ventricular
RVFWSL: Right ventricular free wall longitudinal strain
2DSTE: Two-dimensional speckle-tracking echocardiography
SBT: Spontaneous breathing trial
SpΟ_2_: Arterial oxygen saturation measured by pulse oximetry
PaO_2_: Partial pressure of oxygen
PaCO_2_: Partial pressure of carbon dioxide
SaO_2_: Hemoglobin oxygen saturation
*f*: Respiratory frequency
*V*_T_: Tidal volume
*V*_E_: Minute ventilation
PEEP: Positive end-expiratory pressure
